# A novel Cdk9 inhibitor preferentially targets tumor cells and synergizes with fludarabine

**DOI:** 10.18632/oncotarget.1568

**Published:** 2013-12-18

**Authors:** Elisabeth Walsby, Guy Pratt, Hao Shao, Abdullah Y. Abbas, Peter M. Fischer, Tracey D. Bradshaw, Paul Brennan, Chris Fegan, Shudong Wang, Chris Pepper

**Affiliations:** ^1^ Cardiff CLL Research Group, Institute of Cancer & Genetics, School of Medicine, Cardiff University, Heath Park, Cardiff, UK; ^2^ CRUK Institute for Cancer Studies, University of Birmingham, Birmingham, UK; ^3^ School of Pharmacy and Centre for Biomolecular Sciences, University of Nottingham, Nottingham, UK; ^4^ School of Pharmacy and Medical Sciences, University of South Australia, Adelaide, Australia

**Keywords:** CLL, cdk9, synergy, MCL1

## Abstract

Cdk9 is a key elongation factor for RNA transcription and functions by phosphorylating the C-terminal domain of RNA polymerase II. Here we present direct evidence that cdk9 is important for cancer cell survival and describe the characterization of the potent cdk9 inhibitor CDKI-73 in primary human leukemia cells. CDKI-73 induced caspase-dependent apoptosis that was preceded by dephosphorylation of cdk9 and serine 2 of RNA polymerase II. CDKI-73 was more potent than the pan-cdk inhibitor flavopiridol and showed >200-fold selectivity against primary leukemia cells when compared with normal CD34+ cells. Furthermore, CDKI-73 was equipotent in poor prognostic sub-groups of leukemia patients and showed cytotoxic synergy with the nucleoside analog fludarabine. The Mechanism of synergy was associated with CDKI-73-mediated transcriptional inhibition of MCL1 and XIAP that was maintained when used in combination with fludarabine. Our data present a strong rationale for the development of cdk9 inhibitors such as CDKI-73 as anticancer therapeutics.

## INTRODUCTION

A number of human cancers, including chronic lymphocytic leukemia (CLL), are associated with the over expression of anti-apoptotic BCL2 family proteins [1–3]. CLL is the most common leukemia in the western world and accounts for almost half of all leukemias in older adults [4]. It is characterized by the progressive accumulation of monoclonal CD5+ B-cells in lymphoid tissues, bone marrow and peripheral blood and by the resistance of neoplastic cells to apoptosis [5]. The most challenging aspect of the management of CLL is the treatment of relapsed patients [6] and given that most CLL patients who require treatment will develop resistance to conventional drugs, the identification of new CLL therapies remains a high priority.

The molecular mechanisms that underpin drug resistance in CLL cells are likely to be complex, but the increased expression of anti-apoptotic proteins clearly contributes to this process[3]. MCL1, a member of the BCL2 family, is particularly associated with chemo-resistance and poor prognosis [7, 8] suggesting that therapeutic strategies targeting this protein may be of particular value in CLL. Given the short half-life of MCL1 [9], one strategy for targeting MCL1 is transient inhibition of transcription.

Transcription initiation and elongation is regulated by the cyclin dependent kinases (cdk7 and cdk9), which phosphorylate the carboxy-terminal domain (CTD) of RNA polymerase II [10]. Cdk9 and its cyclin partner, cyclin T1, are highly expressed in CLL suggesting that it may play a role in the pathology of this disease [11, 12]. Indeed, inhibition of these cdks by the cdk inhibitors flavopiridol, SNS-032 and R-roscovitine results in rapid depletion of MCL1 and the induction of apoptosis in primary CLL cells [13–15]. Although these pan cdk inhibitors showed great promise in pre-clinical models, they have proven to have a narrow therapeutic window in clinical trials, with complications relating to scheduling and administration and significant side effect profiles [16–18]. We therefore initiated a program of drug development to identify cdk9 inhibitors with more favorable drug-like properties and an improved therapeutic index.

Here we provide proof-of-concept that cdk9 plays a key role in tumor cell survival as selective cdk9 inhibition. using an shRNA strategy, triggered CLL cell apoptosis confirming cdk9 as a potential anti-cancer therapeutic target. We also describe the preclinical evaluation of the potent cdk9 inhibitor, CDKI-73 in primary CLL cells and detail its potential as a clinical agent both as a single agent and in combination with the purine nucleoside analog fludarabine.

## RESULTS

### Cdk9 knockdown inhibits survival of MEC-1 cells and primary CLL cells

**Figure 1:**
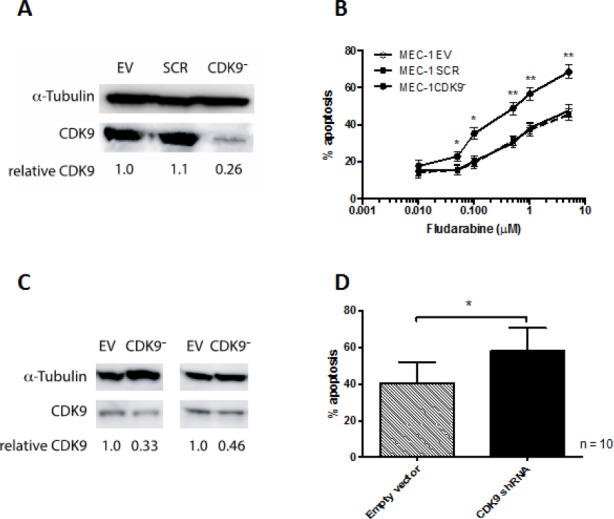
Cdk9 knock-down in MEC-1 cells and primary CLL cells In order to establish whether cdk9 is a valid therapeutic target, lentivirus containing cdk9 shRNA was produced. In the first instance (A) MEC-1 cells were infected with virus and following puromycin selection cells were cultured for 48h and cell viability assessed. (B) MEC-1 cells transfected with cdk9 shRNA showed significantly increased spontaneous apoptosis when compared with empty vector and scrambled sequence controls. (C) Next primary CLL cells were subjected to the same lentiviral infection protocol. Cdk9 was knocked down to approximately 50% of the empty vector control levels in these cells and this resulted in (D) a significant increase in spontaneous apoptosis. *P < 0.05, **P < 0.0001.

As a first step, we set out to establish the biological consequences of specifically inhibiting cdk9. We silenced cdk9 expression using a lentiviral short hairpin RNA (shRNA) construct in the immortalized CLL cell line, MEC-1 [19] and in primary CLL cells freshly isolated from patients. Cdk9 silencing was most effective in the MEC-1 cell line (Figure 1A) and stable knockdown was possible in these cells to 20 % of empty vector controls. Furthermore, cdk9- MEC-1 cells showed significantly increased sensitivity to fludarabine when compared to empty vector and scrambled vector controls (Figure 1B). Despite the technical challenges associated with genetic modification of primary CLL cells, short-term knockdown of cdk9 was also achieved in primary CLL cells to approximately 50% of empty vector controls (Figure 1C) and this resulted in a significant increase in spontaneous apoptosis (Figure 1D). Taken together, these results demonstrate that cdk9 inhibition has a biological consequence in CLL cells and confirm that cdk9 inhibition is a valid anticancer therapeutic strategy.

### The novel cdk9 inhibitor, CDKI-73, shows preferential cytotoxicity in CLL cells

**Figure 2:**
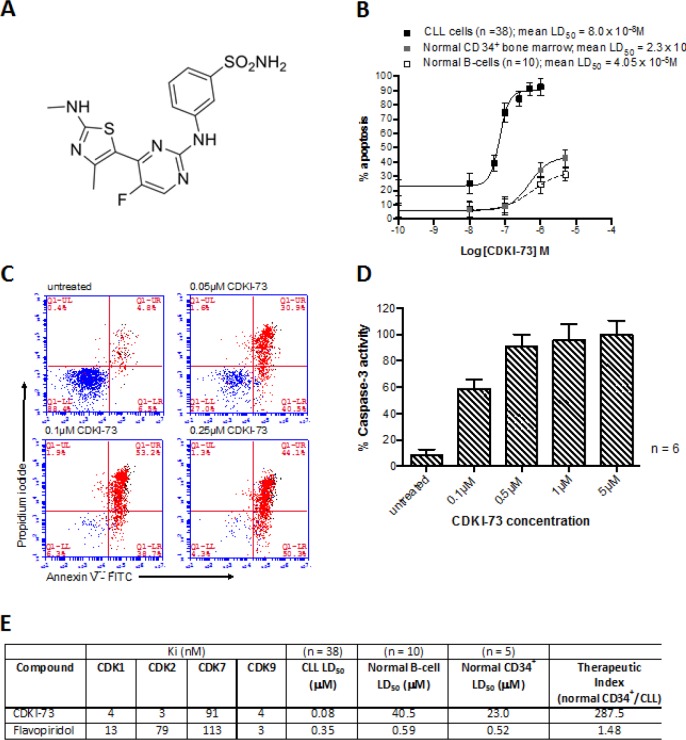
CDKI-73 shows selective toxicity to CLL cells (A) Chemical structure of 3-(5-fluoro-4-(4-methyl-2-(methylamino)thiazol-5-yl)pyrimidin-2-ylamino)benzenesulfonamide (CDKI-73) (B) Shows an example of overlaid sigmoidal dose-response curves for CDKI-73 in CLL cells, normal B-cells and normal CD34+ bone marrow. All cells were cultured in the presence of increasing concentrations of CDKI-73 for 48h. (C) CDKI-73 caused a dose-dependent increase in Annexin V positive cells and this was preceded by (D) a dose-dependent increase in caspase-3 activation after 24h in culture. (E) Table of the kinase inhibition profiles for CDKI-73 in comparison with flavopiridol together with LD50 values in primary CLL cells, normal B-cells and normal CD34+ bone marrow.

We have recently reported the development of a novel class of 5-substituted 4-(thiazol-5-yl)-2-(phenylamino)pyrimidines with cdk9 inhibitory activities [20, 21]. These inhibitors specifically target the cdk9-ATP gatekeeper residue Ph30 and ribose-binding pocket and structure-activity relationship analysis revealed the importance of the 5C-group of pyrimidine core for cdk9 potency and selectivity [21]. Here, we report the biological evaluation of our lead compound CDKI-73 (Figure 2A) that was selected on the basis of its biological potency and its excellent pharmacological properties. It exhibited a favorable pharmacokinetic profile with oral bioavailability of F = 56% following a single intravenous bolus dose at 2 mg/kg and an oral dose at 10 mg/kg in mice. All of the in vitro analyses were performed using primary CLL cells and the pan-cdk inhibitor flavopiridol was used as a comparator. CDKI-73 was cytotoxic to all of the CLL samples tested (n = 38) with a mean LD_50_ value of 0.08μM ± 0.10 μM following exposure to drug for 48h. In contrast, normal B-lymphocytes (n = 10) and CD34+ normal bone marrow cells (n = 5) were significantly less susceptible to the cytotoxic effects of CDKI-73 (Figure 2B). The mechanism of CDKI-73-induced cell killing was confirmed to be apoptosis (Figure 2C) and this was mediated via a dose-dependent induction in caspase-3 activation at an early time point (8h) following exposure to CDKI-73 (Figure 2D). The kinase inhibition profiles of CDKI-73 and flavopiridol were very similar (Figure 2E) but the notional therapeutic indices for the two agents were remarkably different with CDKI-73 showing significantly enhanced selectivity for CLL cells over normal B-lymphocytes and CD34+ bone marrow cells. The apparent discrepancy in these results may be explained, at least in part, by the recent finding that flavopiridol is also a DNA damaging agent [20] and that it induces ER stress and autophagy [22].

### CDKI-73 is equipotent in poor prognostic subsets and retains efficacy under pro-survival co-culture conditions

**Figure 3:**
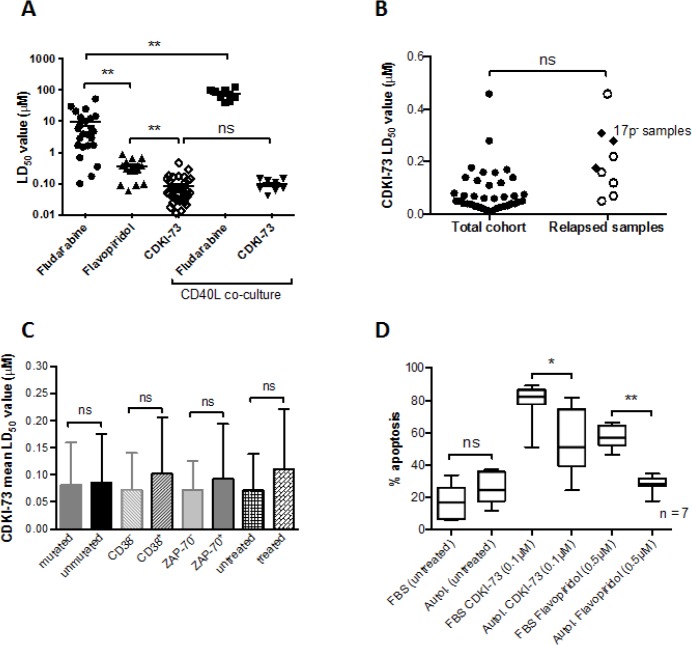
CDKI-73 is more potent than fludarabine and flavopiridol and is equipotent in samples derived from relapsed patients (A) CDKI-73 was significantly more cytotoxic to CLL cells than flavopiridol and fludarabine. (B) CDKI-73 retained potency in samples derived from patients with relapsed disease (n = 7) and in three patient samples with a p53 deletion. (C) CDKI-73 was equipotent in samples derived from prognostic subsets of CLL patients. (D) Given the plasma binding characteristics of flavopiridol, the effects of CDKI-73 were determined in autologous plasma. Although CDKI-73 exhibited reduced potency when compared to cultures carried out in fetal bovine serum (n = 6), it was still able to induce apoptosis in the presence of autologous plasma and was significantly more active than flavopiridol. *P<0.05, **P<0.0001.

The in vitro cytotoxic effects of CDKI-73 were compared with flavopiridol and the purine nucleoside analog fludarabine. Under standard liquid culture conditions CDKI-73 was significantly more potent than flavopiridol and fludarabine (Figure 3A). Furthermore, we compared the in vitro effects of CDKI-73 and fludarabine in parallel co-culture experiments in which primary CLL cells were co-cultured with CD40L-expressing mouse embryonic fibroblasts; conditions known to be highly cytoprotective to CLL cells [23]. CDKI-73 retained cytotoxicity under these conditions whereas the cytotoxic effects of fludarabine were abrogated. Importantly, CDKI-73 was equipotent in CLL cells derived from patients who had suffered clinical relapse following standard chemotherapy including three samples with a 17p deletion (Figure 3B). In keeping with this finding, when we broke the CLL cohort down into prognostic subsets, none of the poor prognostic markers appeared to significantly impact upon the ability of CDKI-73 to induce apoptosis (Figure 3C). All of the data presented in this manuscript were derived from culture conditions supplemented with 10% fetal bovine serum. However, given the unfavorable human plasma binding characteristics of flavopiridol [24], we compared the cytotoxic effects of CDKI-73 in culture conditions supplemented with 10% autologous human plasma. As expected, the addition of autologous plasma to flavopiridol-treated cultures resulted in a marked reduction in apoptosis (>2-fold) in all the samples tested (n = 7). Although CDKI-73 also showed reduced cytotoxicity under these conditions, the effects were much less pronounced (Figure 3D) suggesting that this agent might have superior bioavailability when compared to flavopiridol.

### CDKI-73 inhibits the phosphorylation of serine 2 of RNA polymerase II and MCL1 protein expression in CLL cells

**Figure 4:**
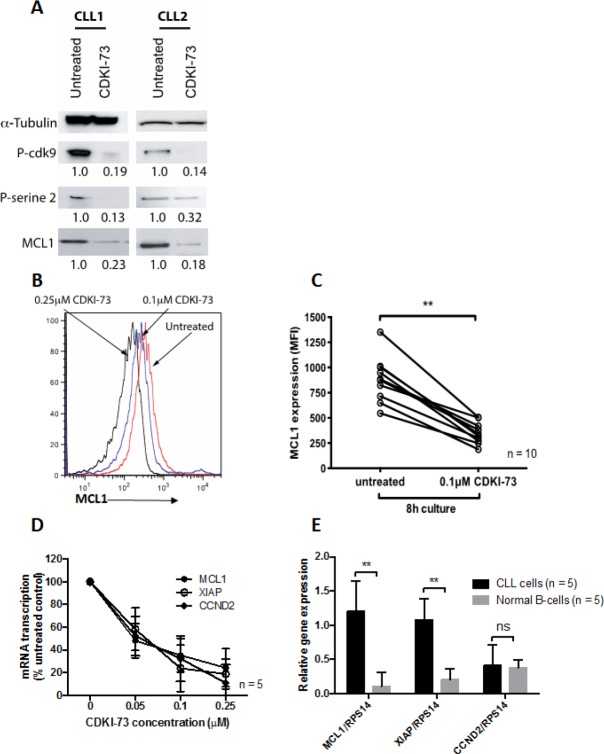
CDKI-73 inhibits cdk9 phosphorylation, RNA polymerase II phosphorylation, MCL1 transcription and protein expression Primary CLL cells were cultured in the presence of 0.1 μM CDKI-73 for 8h. (A) Western blots from two individual patients demonstrating inhibition of cdk9 phosphorylation, RNA polymerase II phosphorylation and MCL1 expression. (B) Overlaid histograms showing CDKI-73-mediated inhibition of MCL1 protein expression (C) MCL1 protein expression was consistently inhibited by CDKI-73 at 8h in all the primary CLL samples tested. Importantly, this change in MCL1 protein expression preceded evidence of apoptosis induction. (D) The inhibition of MCL1 protein was mirrored by the marked inhibition MCL1, XIAP and CCND2 transcription at 4h. (E) Primary CLL cells showed significantly elevated transcription of MCL1 and XIAP when compared to normal B-cells perhaps providing a rationale for the selectivity of CDKI-73 in CLL cells. **P < 0.0001.

We next investigated the molecular mechanisms that underpin the cytotoxic effects of CDKI-73 in CLL cells. Treatment of CLL cells with 0.1 μM CDKI-73 for 4h inhibited the phosphorylation of cdk9 and ser2 of RNA polymerase II (Figure 4A). Phosphorylation of ser2 of RNA polymerase II is essential for RNA polymerase II-dependent transcription[25] and inhibition of this process has been shown to preferentially deplete labile proteins like MCL1. The importance of MCL1 protein expression has been previously shown in CLL and MCL1 depletion is sufficient to induce CLL cell apoptosis[25]. Here we demonstrate that CDKI-73 induces a rapid loss of MCL1 protein (Figures 4A–4C) and this is mediated by significant inhibition at the level of gene transcription (Figure 4D). However, this inhibition is not restricted to MCL1 as similar reductions in XIAP and CCND2 were also observed following exposure to CDKI-73 for 4h (Figure 4D). It is worthy of note that normal B-cells show significantly lower levels of transcription of MCL1 and XIAP compared to CLL cells (Figure 4E) perhaps providing a rationale for the preferential toxicity of CDKI-73 in leukemia cells.

### CDKI-73 synergizes with fludarabine

**Figure 5:**
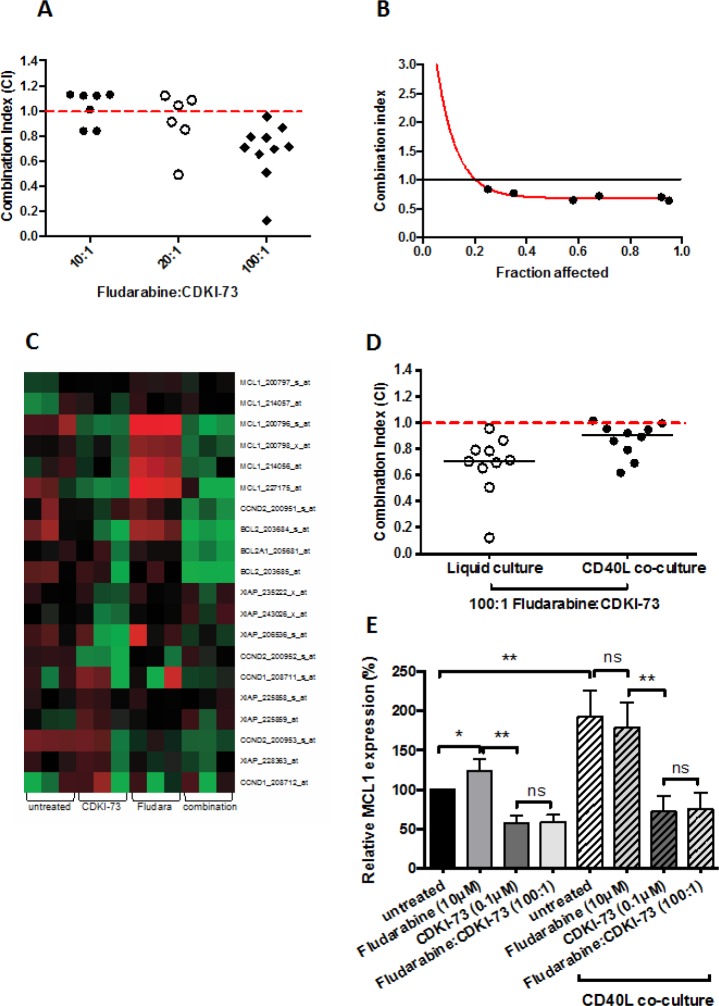
CDKI-73 synergizes with fludarabine even on pro-survival CD40L co-culture (A) Different molar ratios of fludarabine:CDKI-73 were tested on primary CLL cells in 48h cytotoxicity assays. The combination ratios were based on the LD50 values for each drug and the maximum tolerated dose of fludarabine in vivo. 100:1 was shown to give the strongest synergy in all the samples tested. (B) Synergy was observed at all the concentrations of drug combination tested. (C) Gene expression profiling revealed a potential mechanism for the synergy observed. CDKI-73 inhibited the transcription of MCL1, BCL2, XIAP and CCND1 and D2. In contrast, fludarabine induced MCL1, BCL2 and XIAP transcription providing a rationale for the drug resistance that commonly occurs following retreatment with fludarabine. Importantly, the combination of CDKI-73 and fludarabine showed a marked repression of MCL1, BCL2, XIAP and CCND1 and D2. We next tested the effect of the combination under pro-survival, CD40L-expressing co-culture conditions. (D) These conditions are known to induce marked resistance to fludarabine but synergy was retained when used in combination with CDKI-73. (E) Finally we used QRT-PCR to assess the relative expression of MCL1 under the various drug conditions with and without co-culture with CD40L-expressing mouse fibroblasts. In keeping with our gene expression profiling data, CDKI-73 repressed MCL1 both alone and in combination with fludarabine under all conditions. In contrast fludarabine induced MCL1, which was maintained under co-culture conditions. *P<0.05, **P< 0.0001.

Fludarabine-based treatment options are currently the standard of care for CLL patients without significant comorbidities. We therefore assessed the in vitro effects of combining CDKI-73 with fludarabine. The fixed molar ratio used in this study was determined experimentally. The ratios tested were constrained by the maximum clinically achievable dose of fludarabine and the relative potency of each agent. The most synergistic interaction was achieved using a fixed molar ratio of 100:1 (fludarabine:CDKI-73) (Figure 5A). All of the samples tested (n = 10) showed synergy, with a median combination index of 0.71. Furthermore synergy was observed over the range of concentrations of drug used as shown in Figure 5B. In an attempt to understand the underlying molecular mechanisms for the synergy observed, we performed gene expression profiling of CLL cells treated with 10 μM fludarabine, 0.1 μM CDKI-73 and the combination of the two agents (100:1). Gene expression changes were observed under all drug treatment conditions when compared with untreated controls. Figure 5C shows the relative expression changes in genes known to be susceptible to RNA polymerase II inhibition. CDKI-73 as a single agent down regulated MCL1, XIAP and CCND1 and CCND2. In contrast, fludarabine induced the expression of MCL1 but this induction was reversed by the combination of CDKI-73 and fludarabine. Clearly other molecular mechanisms may also contribute to the synergy observed between these agents but the suppression of MCL1, XIAP and CCND1 and CCND2 seem likely to be major factors.

### CDKI-73 remains synergistic with fludarabine under pro-survival co-culture conditions

We have previously shown that co-culture of primary CLL cells with CD40L-expressing mouse embryonic fibroblasts completely abrogates the cytotoxic effects of fludarabine [23]. We therefore set out to determine whether the addition of CDKI-73 could reverse the drug resistance to fludarabine observed under these conditions. Although the combination index increased (median = 0.92), 8/10 samples tested showed cytotoxic synergy and the other two additive effects under these pro-survival culture conditions (Figure 5D). Analysis of the changes in transcription of MCL1 under these conditions confirmed that co-culture significantly induced MCL1 both in untreated CLL cells and cells exposed to fludarabine. In contrast, CDKI-73 significantly inhibited MCL1 transcription as a single agent and in combination with fludarabine (Figure 5E).

## DISCUSSION

Cancer cells often appear to demonstrate oncogene addiction for anti-apoptotic proteins in order to maintain their survival advantage and resist apoptosis. One such cancer is chronic lymphocytic leukemia (CLL) in which over expression of BCL2 family proteins is a hallmark of the disease. [3] The expression of MCL1 is particularly associated with inferior clinical outcomes in this condition[7, 26–28]; a protein with a short half-life at both the mRNA and protein levels [9]. Cdk9 is a key regulator of RNA Polymerase II elongation and hence is particularly critical to the maintenance of expression of short-lived proteins [29]. Given the clear clinical importance of MCL1 in CLL, we rationalized that targeted inhibition of cdk9 might prove to be a useful therapeutic strategy in CLL and other cancers in which MCL1 is over expressed[30]. In the first instance, we tested our working hypothesis by silencing cdk9 using an shRNA approach. Our data provide the first direct evidence that specific cdk9 inhibition can alter the survival characteristics of both an immortalized leukemia cell line and primary leukemia cells derived from patients. This new information provides a strong rationale for the development of small molecule inhibitors targeting cdk9.

In order to develop cdk9 inhibitors, we designed a series of substituted 4-(thiazol-5-yl)-2-(phenylamino) pyrimidine derivatives with functional groups attached to the 5C-pyrimidine or 4C-thiazol ring moiety [20, 21, 31]. These compounds target the cdk9-ATP gatekeeper residue Ph30 and ribose-binding pocket thereby conferring increased selectivity. In this paper we describe the biological characterization of one of our lead compounds, CDKI-73. This compound has potent cdk9 inhibitory activity and excellent pharmacological properties. Intriguingly, it has a similar cdk inhibitory profile to flavopiridol but manifests a remarkably different toxicity profile in primary CLL cells when compared to normal B-lymphocytes and normal CD34+ bone marrow cells; CDKI-73 is more than 200 times more selective than flavopiridol against CLL cells. We hypothesize that this is caused, at least in part, by the off target DNA damaging properties of flavopiridol that we recently reported[20].

At the molecular level, CDKI-73 inhibited the phosphorylation of cdk9 and ser2 residue of RNA polymerase II. This resulted in the inhibition of MCL1 protein expression which we confirmed using both Western blotting and flow cytometric quantification. This reduction in protein expression was mirrored by the inhibition of MCL1 transcription and consistent with the inhibition of RNA polymerase II, we demonstrated similar reductions in the transcription of CCND2 and XIAP at the same early time point.

In addition to the promising potency and selectivity manifested by CDKI-73, we demonstrate here that it retains efficacy in primary CLL samples derived from poor prognostic subsets including those who had relapsed following fludarabine-based regimens. Given that most CLL patients respond to frontline therapy but later relapse [32], there is a clear need for novel therapeutics that can be used in this setting. It is worthy of note that p53 mutation/deletion is more prevalent in relapsed patients [33] and here we show that CDKI-73 has similar potency in this context. Furthermore, CDKI-73 appears to have more favorable plasma binding characteristics than flavopiridol. Although replacement of FBS with autologous plasma resulted in the reduction in potency of CDKI-73, it was much less marked than that observed with flavopiridol.

Given that fludarabine-based regimens still dominate the treatment of CLL [34–36], we assessed the effects of combining fludarabine with CDKI-73. We were able to demonstrate synergy between these two agents consistent with their distinct mechanisms of action. Furthermore, this was explored at the molecular level using gene expression profiling. As we predicted, CDKI-73 inhibited MCL1, BCL2, XIAP and CCND1 and CCND2. In contrast, fludarabine appeared to induce the expression of MCL1 and XIAP offering a potential explanation for the in vitro and in vivo drug resistance that often ensues following exposure to fludarabine [23, 33]. The combination of CDKI-73 with fludarabine reversed the fludarabine-mediated increase in MCL1 and XIAP providing a molecular rationale for the cytotoxic synergy observed. Furthermore, synergy was retained between these two agents even under pro-survival co-culture conditions. Fludarabine is largely ineffective under these conditions [23] (Figure 3A) indicating that CDKI-73 is capable of reversing fludarabine resistance. At the molecular level our data strongly suggest a role for MCL1-mediated fludarabine resistance and so the ability of CDKI-73 to deplete the expression of this protein would appear to be key. Taken together, we provide compelling evidence that targeting cdk9 in CLL represents a promising therapeutic strategy both as a single agent and in combination with fludarabine.

## METHODS

### Cell isolation and culture

Peripheral blood samples from CLL patients (n = 38), normal age-matched peripheral blood (n = 10) and normal CD34+ bone marrow samples (n = 5) were obtained in accordance with the ethical approval obtained from South East Wales Research Ethics Committee (02/4806). Mononuclear cells were separated using Ficoll-Hypaque (Sigma, Poole, UK) and autologous plasma was collected for use in some experiments. CLL B-cells and normal B-cells were purified by negative selection using CD3 microbeads. CD34+ bone marrow cells were purified by positive selection using CD34+ microbeads. All purification proceedures were carried out on an AutoMACS Pro separator (Miltenyi Biotec, Bisley, UK). Subsequently, 1×106/ml cells were maintained in RPMI medium supplemented with 10% fetal bovine serum (FBS), penicillin (50U/ml), streptomycin (50μg/ml) and recombinant human IL-4 (R and D Systems, Abingdon, UK) (5ng/ml). Normal Mouse embryonic fibroblast L-cells, either non-transfected (NTL) or L-cells expressing CD40 ligand (CD40L)[37] were used where indicated as feeder layers.

### Chemical analysis

Synthesis of a novel class of 5-substituted 4-(thiazol-5-yl)-2-(phenylamino)pyrimidines have been recently described[21]. 3-(5-Fluoro-4-(4-methyl-2-(methylamino)thiazol-5-yl)pyrimidin-2-ylamino) benzenesulfonamide (CDKI-73) was obtained from 3-guanidinobenzenesulfonamide and 3-(dimethylamino)-2-fluoro-l-(4-methyl-2-(methylamino)thiazol-5-yl) prop-2-en-l-one. mp 268 - 270 °C. Anal. RP-HPLC: tR 11.45 min, purity 99 %. 1H-NMR (DMSO-d6): δ 2.88 (d, 3H, J = 4.8 Hz, CH3), 7.29 (s, 2H, NH2), 7.40 (d, 1H, J = 8.0 Hz, Ph-H), 7.47 (t, 1H, J = 8.0 Hz, Ph-H), 7.89 (d, 1H, J = 8.0 Hz, Ph-H), 8.13 (br q, 1H, J = 4.8 Hz, NH), 8.25 (s, 1H, Ph-H), 8.47 (d, 1H, J = 3.2 Hz, Py-H), 9.83 (s, 1H, NH). 13C-NMR (DMSO-d6): δ 19.43 (d, J = 5 Hz), 31.33, 109.97 (d, J = 8 Hz), 115.81, 118.78, 121.89, 129.51, 141.46, 144.94, 145.97 (d, J = 25 Hz), 147.63 (d, J = 12 Hz), 147.94 (d, J = 248 Hz), 155.66, 156.04, 171.34. HR-MS (ESI+): m/z [M + H]+ calculated for C15H16FN6O2S2, 395.0760, found 395.0641. Flavopiridol and fludarabine were purchased from Sigma-Aldrich (Poole, UK).

### Lentiviral production and transduction of stable cell lines

Bacterial glycerol stocks containing the lentiviral plasmid vector pLKO. 1-puro with shRNA inserts against CDK9 (495) along with an empty vector (EV) and a scrambled shRNA (SCR) controls were obtained from Sigma Aldrich (Poole, UK). For lentiviral production, 293T cells were transfected with complexes comprising 1μg lentiviral shRNA plasmid (495, EV or SCR), 0.75 μ*g* P8Δ91 plasmid, and 0.5 μg pMD2G plasmid using the Effectene reagent (Qiagen) according to the manufacturer's instructions. Transfected 293T cells were incubated at 37oC for 48h before the resulting lentiviral particles were harvested by centrifugation and concentrated using the Clontech Lenti-X concentrator kit (Lonza, Wokingham, UK). Concentrated virus was added to MEC-1 cells and incubated for 48h. Lentivirus-transduced cells were then selected by addition of puromycin (1 μg/ml) to the culture for two weeks. Subsequently, the relative sensitivity to fludarabine of EV, SCR and 495 transduced cells was assessed by flow cytometry.

### Lentiviral modulation of cdk9 in primary CLL cells

Primary CLL cells were incubated with the transfected 293T cells for 48h before cell viability was measured and protein harvested for immunoblotting.

### Apoptotic effects of CDKI-73 and fludarabine on primary CLL cells

Cells were treated with CDKI-73 (0-1 μM) for 48h before cell viability was determined by flow cytometry using Annexin V and propidium iodide as previously described[23]. In parallel experiments CLL cells were also treated with 0.1 μM CDKI-73 for 4h and cells were harvested for protein extraction and subsequent immunoblotting.

### Protein isolation and immunoblotting

CLL cells were washed with PBS and lysed by resuspension in lysis buffer (HEPES 50 mM, sodium fluoride 5 mM, iodoacetamide 5 mM, sodium chloride 75mM, NP40 1%, PMSF 1 mM, sodium orthovanadate 1 mM, protease inhibitors (Sigma) 1%, phosphatase inhibitor cocktail 2 (Sigma) 1%, phosphatase inhibitor cocktail 3 (Sigma) for 30 minutes at 4oC followed by centrifugation at 16 000 × g. Clarified lysates were subjected to electrophoresis using NuPage precast 4–12% Bis-Tris gels (Invitrogen, Paisley, UK) followed by transfer to PVDF membranes (GE Healthcare UK Ltd. Little Chalfont, UK). Immunoblotting was performed with antibodies against cdk9, tubulin (Abcam, Cambridge, UK), phospho-cdk9, MCL1 (New England Biolabs, Hitchin, UK) and RNA polymerase II phospho-ser2 (Active Motif, Rixensart, Belgium).

### Determination of synergy between cdk9 inhibitors and fludarabine

CDKI-73 was combined with fiudarabine at an experimentally determined fixed molar ratio of 100:1 (fludarabine:CDKI-73). CLL cells were treated with both cdk inhibitors and fiudarabine alone and in combination to determine whether there were synergistic interactions between the two agents. Synergy was calculated according to the Chou and Talalay median effect method[38].

### Real-time reverse transcription-PCR

Untreated cells and cells treated with CDKI-73, fiudarabine or their combination (fiudarabine: CDKI-73, 100:1) for 4h 5×106 CLL cells were re-suspended in 1ml Trizol reagent and RNA was extracted using chloroform and isopropanol. RNA (1μg) was used in a 20μL reverse transcription (RT) reaction[23]. SYBR Green technology (Roche Diagnostics, Burgess Hill, UK) was used to quantify the amount of RNA present in each sample using primer pairs for CCND2 (cyclin D2), MCL1, XIAP and RPS14. All primers were purchased from Eurogentec Ltd (Southampton, UK). The amount of mRNA was assessed using real-time RT-PCR using the LightCycler System (Roche Diagnostics). The amount of RPS14 mRNA was quantified in all samples as an internal house-keeping control, and the results of the real-time RT-PCR were expressed as normalized target gene values (e.g. the ratio between MCL1 and RPS14 transcripts calculated from the crossing points of each gene). All experiments were performed in duplicate. Total RNA was amplified using the following primers:CCND2: 5′- tcattgagcacatccttcgcaagc-3′ (forward) and 5′- ggcaaacttgaagtcggtagcaca-3′ (reverse);MCL1: 5′-aaaagcaagtggcaagagga-3′ (forward) and 5′-ttaatgaattcggcgggtaa-3′ (reverse);XIAP: 5′-tgggacatggatatactcagttaacaa-3′(forward) and 5′-gttagccctcctccacagtgaa-3′ (reverse);RPS 14: 5′-ggcagaccgagatgaactct-3′ (forward) and 5′-ccaggtccaggggtcttggt-3′ (reverse).

### Microarray procedures

The detailed protocol for sample preparation and microarray processing is available from Affymetrix (http://www.affymetrix.com). Briefly, total RNA was extracted from CLL cells treated with 0.1 μM CDKI-73, 10μM fiudarabine or the two drugs in combination for 4h. First strand complementary DNA (cDNA) was synthesized from 5 μg total RNA using a T7-(dT)24 primer (Genset Corp, San Diego, CA, USA) and reverse-transcribed with the Superscript Double-Stranded cDNA Synthesis Kit (Invitrogen Life Technologies, San Diego, CA, USA). After second strand synthesis, the resulting cDNA was subjected to an in vitro transcription reaction using a Bioarray kit (Enzo Diagnostics, New York, NY, USA) to generate biotinylated cRNA. This was subsequently fragmented and hybridized to the Affymetrix U133 2.0 Gene Chips. After hybridization, each microarray was washed, stained and scanned with an argon-ion confocal laser, with excitation at 488 nm and detection at 570 nm. The data discussed in this publication have been deposited in NCBI's Gene Expression Omnibus and are accessible through GEO Series accession number GSE48258 (http://www.ncbi.nlm.nih.gov/geo/query/acc.cgi?acc=GSE48258).

### Statistical Analysis

The significance of differences between experimental conditions was determined using the Student's t-test for paired and unpaired observations. All data was confirmed as Gaussian or a Gaussian approximation using the omnibus K2 test. To assess the interaction between agents, the median effect method was employed using CalcuSyn software (CalcuSyn; Biosoft International, Ferguson, MO). The combination index was calculated for the two-drug combination using an experimentally determined fixed concentration ratio. Combination index values < 1.0 indicated a synergistic interaction. Affymetrix gene expression data were processed using MADRAS software (Developed by Peter Giles, Cardiff University). P values < 0.05 were considered significant.
